# Impact of SARS-CoV-2 on Provided Healthcare. Evidence From the Emergency Phase in Italy

**DOI:** 10.3389/fpubh.2020.583583

**Published:** 2020-11-23

**Authors:** Rossella Di Bidino, Americo Cicchetti

**Affiliations:** ^1^Health Technology Assessment Unit, Fondazione Policlinico Universitario Agostino Gemelli Istituto di Ricovero e Cura a Carattere Scientifico, Rome, Italy; ^2^Graduate School of Health Economics and Management (Alta Scuola di Economia e Management dei Sistemi Sanitari), Universitá Cattolica del Sacro Cuore, Rome, Italy

**Keywords:** COVID-19, SARS-CoV2, cancer care, cardiology, Health Services Research, Response model (RM)

## Abstract

The SARS-CoV-2 (COVID-19) pandemic led to an emergency scenario within all aspects of health care, determining reduction in resources for the treatment of other diseases. A literature review was conducted to identify published evidence, from 1 March to 1 June 2020, regarding the impact of COVID-19 on the care provided to patients affected by other diseases. The research is limited to the Italian NHS. The aim is to provide a snapshot of the COVID-19 impact on the NHS and collect useful elements to improve Italian response models. Data available for oncology and cardiology are reported. National surveys, retrospective analyses, and single-hospital evidence are available. We summarized evidence, keeping in mind the entire clinical pathway, from clinical need to access to care to outcomes. Since the beginning, the COVID-19 pandemic was associated with a reduced access to inpatient (−48% for IMA) and outpatient services, with a lower volume of elective surgical procedures (in oncology, from 3.8 to 2.6 median number of procedures/week). Telehealth may plays a key role in this, particularly in oncology. While, for cardiology, evidence on health outcome is already available, in terms of increased fatality rates (for STEMI: 13.7 vs. 4.1%). To better understand the impact of COVID-19 on the health of the population, a broader perspective should be taken. Reasons for reduced access to care must be investigated. Patients fears, misleading communication campaigns, re-arranged clinical pathways could had played a role. In addition, impact on other the status of other patients should be mitigated.

## Introduction

The first autochthonous confirmed case of SARS-CoV-2 (Severe Acute Respiratory Syndrome Coronavirus 2) was registered in Italy on 21 February 2020 in Codogno (Lombardy), generating the first relevant outbreak of the virus. The Italian government ordered a nationwide lockdown, which was effective starting 9 March 2020, while the World Health Organization (WHO) declared COVID-19 a pandemic on 11 March.

At the time this was written, Italy has counted more than 243,000 confirmed cases of COVID-19 and a cumulative number, >195,000, COVID-19 discharged patients. At the peak of the first phase of the emergency, saturation in ICUs reached 54.9 % (1).

Therefore, on addition to promoting measures that limited the transmission of the disease, in-hospital clinical activities had to rapidly adapt their methods of organization to the healthcare emergency. The reduction, or even interruption, of non-COVID related activities were the main solutions suggested to all Regional Health Authorities by the Ministry of Health, with guidelines for the re-organization of services issued on 16 March 2020 (2). Furthermore, prioritization criteria were defined for access to critical services, such as surgical procedures requiring a longer stay in intensive care.

The Graduate School of Health Economics and Management (ALTEMS) has been monitoring the response of the Italian NHS since the beginning of the emergency with a weekly Instant Report (3). The aim is to provide an integrated analysis of available data on COVID-19. The major goals are to identify differences and analogies among national and regional COVID-19 models of care and anticipate short and long term needs. Due to this, since end of May 2020, a specific section is dedicated to the collection and analysis of data regarding the impact of COVID-19 on the care provided to other patients.

During the first phase of the emergency, resources were focused on dealing with the COVID-19 impact on human and organizational resources of the NHS and on making the emergency sustainable. ALTEMS estimated a reduction of more than 860,000 hospital discharges during the COVID-19 outbreak, on the basis of a simulation that took into account hospital discharge data in 2018. During this period, hospital activity was limited to urgent treatments, and we have estimated that more than 520,000 surgeries were not performed during a 4 months period (3) (Instant Report # 9). At the beginning of the so-called Phase 2, it was time to find a new equilibrium between new and “old” healthcare needs. The NHS had to provide assistance both to COVID-19 patients but also to patients affected by other diseases whose needs were put on a sort of waiting list from February until May 2020. In order to better describe this additional burden of care and the consequence of the temporary reorganization of the NHS, the Altems working group is conducting a literature review focused on published studies for the Italian context.

## Methods

A realist literature review (4) was adopted due to the fast evolving scenario. It was conducted to address the following questions: (a) How did the COVID-19 pandemic impact on the care provided to other patients (e.g., patients with non-COVID related clinical conditions) in Italy?; (b) How does it impact on different specialities and level of care in the Italian NHS?; (c) What are the implications in the organization of the NHS at national, regional, and local levels?

The search strategy and keywords were organized around the following three broad realist concepts:

- Context: the activity of the Italian NHS during the emergency phase (from mid-February 2020 to June 2020) of the COVID-19 pandemic;
- Clinical areas: a step-by-step approach was adopted. The research first focused on cardiology and oncology. It will then be extended to transplantation, gastroenterology, nephrology, and so on. The choice of these two areas was pragmatic and based on first available evidence and different levels of care and need involved. For instance, our analysis will provide input on how COVID-19 impacts the ability of the NHS both in responding to urgent need (due to cardiovascular emergencies), as well as providing elective surgical procedures and outpatient care (to cancer patients);
- Impact: healthcare service usage data, measurable health outcomes, and NHS organization is the main focus. Despite the fact that the majority of evidence comes from healthcare providers, it was decided, when available, to include patient perspectives.

At the end of May 2020, a search was conducted on Pubmed, websites of major Italian medical associations, and national medical news websites (such as: Quotidiano Sanità, Il Sole24Ore Sanità, and so on).

All English and Italian language papers published on scientific journals or studies from which reports were published online on reliable websites from February 2020 until the end of May 2020 were included in our review.

## Results

### Selected Papers

A total of 20 studies were selected: five provided data on cardiology, while 15 referred to oncology in Italy. As shown in Table 1, studies on the impact of COVID-19 in cardiology mainly focus on coronary syndrome (ACS). The distribution of studies among NHS levels (national, regional, hospital) is similar for the two clinical areas. Eight out of 20 studies have been performed on the basis of data collected on a national level. Six of the eight studies with a national perspective were in the area of oncology. One of them (5) provided data on hospital admissions for acute coronary syndrome (ACS) in five (out of 21) regions. It was considered representative given the COVID incidence in those regions.

**Table 1:** Selected studies on cardiology and oncology.

**References**	**NHS level**	**Details**	**Location**	**Primary outcome (or focus)**	**Study designed**	**Covered period**	**Sample size**
**Cardiology**							
De Rosa et al. (6)	National	54 centers	Italy	Patients with AMI admitted to intensive cardiac care units	Online survey open to affiliates of the Italian Society of Cardiology	12–19 March 2019 vs. 2020	937 AMI patients
De Filippo et al. (5)	Multicentre	15 centers located in 5 Regions	Piedmont, Liguria, Lombardy, Emilia Romagna, Lazio	Hospital admissions for ACS	Retrospective analysis	1 January to 19 February + 20 February to 31 March 2019 vs. 2020	2202 ACS patients
Piccolo et al. (7)	Regional	20 PCI centers	Campania	Rates of Percutaneous Coronary Revascularization for Acute Coronary Syndromes	Retrospective analysis	30 January to 26 March, 2020	1,831 PCIs
Cosentino et al. (8)	Hospital		Lombardy	In-hospital pathway for Acute Coronary Syndrome patients	Retrospective analysis	13 March to 9 April, 2020	92 ACS patients
Mazzone et al. (9)	Hospital		Lombardy	Re-organization of a referral center for cardiac electrophysiology (EP)	Retrospective analysis	October–December 2019 vs. January–February 2020 vs. March 2020	
**Oncology**							
Costantini et al. (10)	National	Hospices	Italy	Preparedness for and impact of the COVID-19 pandemic on hospices	Cross-sectional telephone survey	Administered between 11–15 March, 2000	16 Hospices
Indini et al. (11)	National	Head physicians *via* hospital medical oncology ward -Oncologi Medici Ospedalieri (CIPOMO)	Italy	COVID-19 containment measures and diffusion in oncology units and its impact on working activities	Survey	Administered between 12–15 March, 2000	122 Head physicians
Jereczek-Fossa et al. (12)	National	125 Directors from Italian radiation oncology wards, members of the AIRO	Italy	Clinical and outpatient activities, patients and staff management during COVID-19 emergency	Survey	Administered between 6 and 16 April, 2020	125 directors
Torzilli et al. (13)	National	Referral centers for HPB, colorectal, esophago-gastric, and sarcoma/soft-tissue tumors	Italy	Elective oncology surgery	Survey	Before vs. entire period (5 weeks, starting 18 February), and during the week (23–27 March, 2020)	54 referral centers
Progetto (14)	National		Italy	Diagnosis, treatment, and follow-up activity during COVID-19 pandemic	Survey	Administered between 14 and 29 April, 2020	774 patients
Lambertini et al. (15)	National	Perspectives of young oncologists	Italy	Practical suggestions on how to implement cancer care during the COVID-19 outbreak	Editorial		
Casanova et al. (16)	Hospital/ Outpatient care	Patients from a Pediatric oncology unit	Lombardy	Patient perception of COVID-19 epidemic	Survey	Administered between 2 and 5 March, 2000	25 patients were receiving treatment; 25 patients were in follow-up, who had completed their treatment; 25 were healthy peers
Brandes et al. (17)	Regional	Oncology wards	Emilia-Romagna	Patients, healthcare workers, risk-reduction measures, and clinical trials	Survey		12 oncology wards
Campi et al. (18)	Hospitals	3 High-volume academic centers for major uro-oncologic surgery	Piedmont, Lombardy, Tuscany	Classification as high priority, major uro-oncologic surgical procedures	Retrospective analysis	12-mo period (2018 or 2019)	2,387 patients
Balduzzi et al. (19)	Hospital	1 Pediatric transplant and haemato-oncology center	Lombardy	Preventive and control measures	Case study		
Bongiovanni et al. (20)	Hospital	1 Osteo-oncology and rare tumor center	Emilia-Romagna	Report of a multidisciplinary approach	Case study	9 March−17 April, 2000	3,348 screened patients (3% with BM)
Kengli et al. (21)	Hospital	1 Radiation oncology ward	Piedmont	Preventive measures and recommendations	Case study		
Vicini et al. (22)	Hospital	1 Division of breast surgery in a cancer hub center	Lombardy	1st month experience/impact	Case study	March 2020	
Montesi et al. (23)	Hospital	1 Radiation oncology unit	Veneto		Case study	1 February−31 March, 2020	
Pezzulla et al. (24)	Hospital	1 Radiotherapy oncology unit	Molise	Measures to minimize the risk of infection among operators	Case study		

The majority of studies are based on surveys. Data cover the February-March 2020 period, and efforts were made to compare the 2020 scenario with previous years. Only two studies (12, 14) are based on a national survey performed in April 2020.

**Figure 1:**
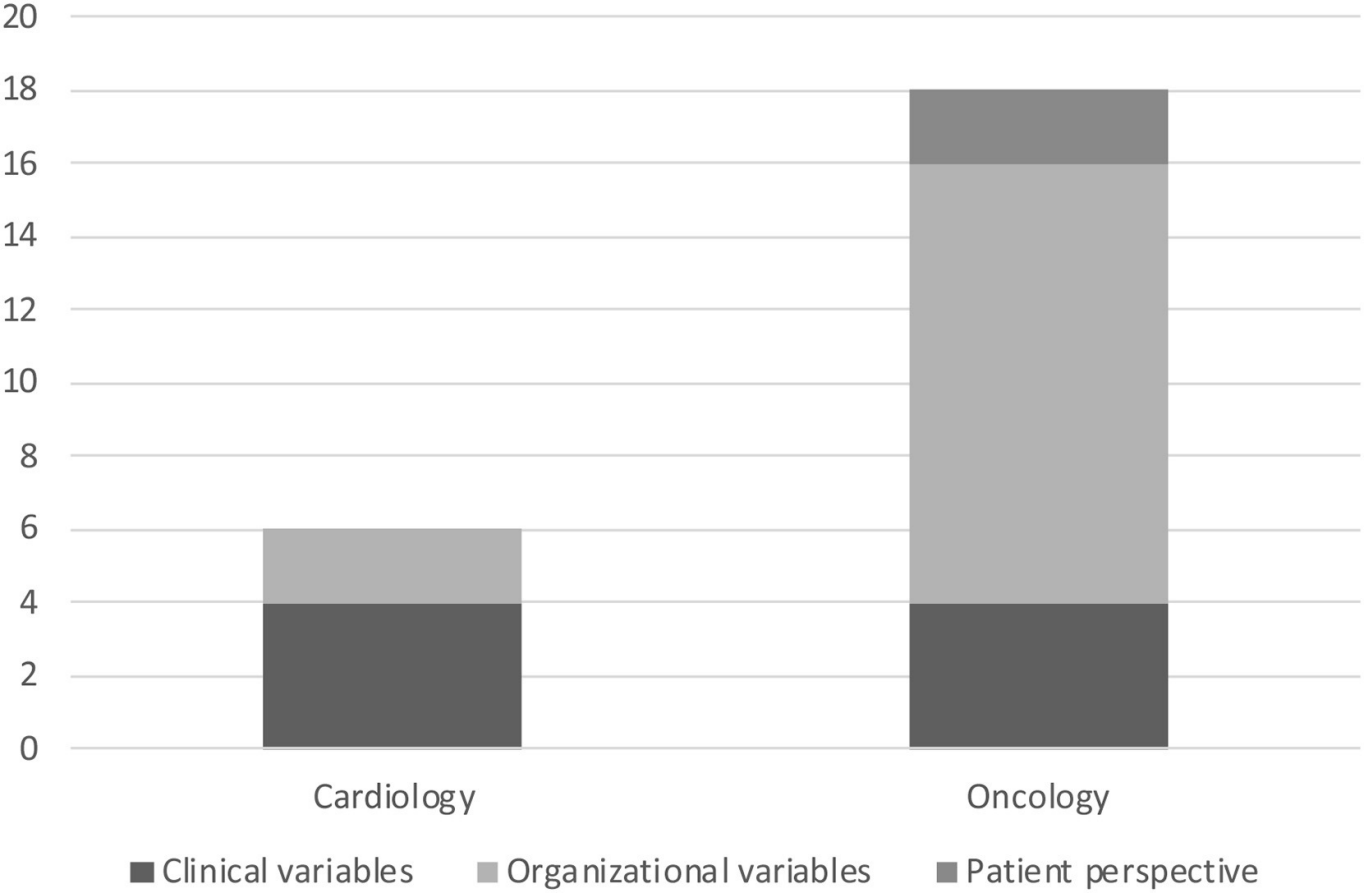
Variables investigated by selected studies.

Selected papers collected data on clinical and/or organizational variables and some of them even on patient perspective. As showed by Figure 1 studies on oncology pay more attention to organizational variables compared to studies on cardiology. While health outcomes, such as mortality, are already available for cardiovascular emergencies (Table 1), the link between hospital re-organization and provision and access to care is investigated more thoroughly for cancer care. For instance, two different surveys on oncology (12, 13) reported how the reduction of available beds (acute and in intensive care) impacted on clinical volumes, surgical procedures, outpatient services, radiation therapy, and so on. In oncology, the first evidence on how telemedicine helped guarantee continuity of care is available (17, 20). In Supplementary Materials, more details are reported on variables/endpoints for which data was collected in selected studies.

Patient perspectives were only investigated in oncology, with two different surveys: the first (16) was limited to adolescent and young adult patients from a pediatric oncology unit in Lombardy, and the other was (14) on a national level. The latter survey, which included 774 responders (Table 1), had the objective of collecting evidence on the impact of the COVID-19 pandemic regarding access to care, from diagnosis to follow up, while the first was based on a total of 75 responders (Supplementary Materials—Table A1) and was more focused on the perception of the risk associated with the outbreak of COVID-19.

### Available Evidence

Table 2 aims to summarize the major consequences of the COVID-19 outbreak on the ability of the NHS to manage cardiologic and oncologic patients. From volumes of activities to continuity of care, the significant negative impact of the pandemic on the operativity of the NHS is clear despite all the initiatives taken at different levels of the NHS.

**Table 2:** Impact of COVID-19 on cardiology and oncology care in Italy.

	**Cardiology**	**Oncology**	**References**
**Volume of activity**			
Overall		*At national level:* In 30.4% of centers, a 10–30% reduction was reported.	(6, 12)
Outpatient		*At national level:* Patients report postponement of follow-up visits (36%). One patient out of five reported cancelation of diagnostic exams.	(14)
Inpatient	*At national level:* 48.4% (95% CI 44.6–52.5) reduction in admissions for AMI.		
Surgical procedures	*At regional level:* 32% decline in the number of PCI for ACS (incidence rate from 178 to 120 cases/100,000 residents).	*At national level*: Number of surgical procedures decreased (median number of 3.8 [IQR 2.7–5.4], per week before COVID-19 emergency, to 2.6 [22–4.4] later).	(7, 13)
Diagnostic exams		*At national level*: Reported limited access to the following hospital facilities: CT in 31% of cases, MRI in 24%, (PET)-CT in 13%, endoscopy in 26%, percutaneous procedures in 20%, endovascular procedures in 15%, and radiotherapy in 11%.	(13)
**Clinical outcomes**			
Mortality	*At national level:* An increase in STEMI case fatality rate [13.7 vs. 4.1% (RR =3.3, 95% CI 1.7–6.6; *P* < 0.001)].		(6)
Complications	*At national level:* An increase in STEMI (RR = 1.8, 95% CI 1.1–2.8; *P* = 0.025) and nSTEMI (RR =2.1, 95% CI 1.05–4.1; *P* = 0.037) patients with major complication.		(6)
**Timing**			
Access to care	*At national level:* 39.2% increase in the time from symptom onset to coronary angiography—AND.	*At regional level:* Follow-up visits were canceled in 16.7% of centers, delayed in 58.3% of centers, and performed by remote assessment in 58.3% medical oncology wards.	(6, 17)
Waiting list	*At national level:* 31.5% increase in the time from first medical contact to coronary revascularization.	*At national level:* In most facilities *(62.4%)*, rescheduling of patient waiting lists (prioritization) was also carried out. Most units (87%) expected to have a median prolongation of 4 weeks in the time interval between the pre-operative multidisciplinary meeting and surgery.	(6, 12)
**Continuity of care**			
Telemedicine		*At national level:* To guarantee the continuity of care, telematic consultations were activated in 78 centers (62.4%).	(13)
		*At regional level:* For a defined group of patients (patients with bone metastases), telemedicine helped in guaranteeing continuity of care and a multidisciplinary approach from first diagnosis to pain management.	(20)
**Research activities**			
Clinical trials		*At regional level:* 66.7% of medical oncology wards suspended accruals of clinical trials.	(17)
**Available resources**			
Beds	*At hospital level:* Internal strategies were adopted for sparing both ICU beds and anesthesiology personnel.	*At national level:* 76% of centers had a reduction in their surgical activity (days of operating room); 83% had less availability of ICU beds; 52 (96%) had a reduction in outpatient clinics.	(9, 13)
Human		*At national level:* > 30% of oncologic structures had to employ their oncologists for guard duties in internal medicine and/or emergency wards; in 23% of cases, guard duties in COVID wards were included.	(11)
		*At national level:* Physicians and RT technicians were most frequently COVID-19 infected, followed by nurses, medical physicists, and other personnel.	(12)
		*At regional level:* COVID-19 infection was diagnosed in 10.1% medical doctors, 5.7% nurses, 11.8% social care workers.	(17)
**Internal organization**			
Hospital	*At hospital level:* Some evidence is available on the adoption of a hub-and-spoke model for cardiology. However, only urgent and non-deferrable procedures were performed.	*At national level:* 85 structures (68%) became COVID-19 centers, requiring an immediate reorganization of the entire facility.	(9, 13)
Ward	*At hospital level:* New, in-hospital pathways for ACS were adopted to guarantee the best and safest treatment for all patients.	*At national level:* 37.5% of RT wards/DHs were converted into COVID-19 centers.	(8, 13)

New clinical pathways were adopted to guarantee patient and personnel safety. At the same time, the optimization of hospital resources (not only ICU beds), and the need to have COVID-19 dedicated personnel, led to a contraction in activities. For instance, 30.4% of oncology centers reported a contraction of their activities of 10–30% (13). The reduction in cardiology was more significant, even for urgent cases, such as AMI patients (48.4% reduction in hospital admissions for AMI).

While initial negative results, in terms of health outcomes, are already available for cardiology, we just registered a clinical relevant reduction in the assistance provide along the entire clinical pathway in oncology.

## Discussions

The COVID-19 outbreak has direct and indirect effects on the healthcare delivery process in the Italian NHS. At national, regional, and local levels, the Italian NHS re-engineered its clinical processes, in order to manage COVID patients, both in hospitals and at home. Nevertheless, the pandemic affected healthcare delivery for non-COVID patients. Our effort was to further emphasize how the COVID-19 emergency had implications for non-COVID patients, along the entire process of care in different settings (hospitals, outpatient services, hospices). Our approach was quite similar to that proposed by Richards et al. (25) to identify all implications on patient pathways in oncology. We collected available data for Italy on COVID-19 implications for diagnosis, surgery, treatment, continuity of care, and research for different clinical areas, as suggested in (25). Our literature review was the first step toward an in-depth analysis of how the healthcare policy implemented (explicitly and implicitly) during the emergency translated into organizational choices adopted at national, regional, and local levels and how it determined short and long term health outcomes.

Based on available data, the re-organization of hospital logistics and clinical activities, during the first phase of the emergency, determined a reduction in inpatient and outpatient services provided to non-COVID patients. In addition, communication activities on the risk of COVID-19 transmission could have contributed to a lower propensity by patients to directly refer to hospitals.

In cardiology, a new organization of the NHS and also patient fears could both explain the lower rate of hospital admission for IMA and ACS (5, 6) and the associated higher case fatality and complication rates, due to a delay in access to care and in diagnosis. The lower number of percutaneous coronary interventions (PCI), especially in women, needs special attention.

In oncology, the reduction of available acute and intensive care beds translated in a lower amount of surgical procedures and was associated with a reduction of outpatient activities. A reported reduction of the overall activity of 10–30% in a third of cancer centers (12) is confirmed by patients. In fact, 36% of those interviewed reported postponement or cancellation of clinical exams and follow up visits (14). The impact of hospital reorganization regarding access to clinical and diagnostic exams, such as CT scans, MRIs, and so on, is not secondary. Even if only one paper (13) provided some evidence on that point, it is a critical step along a clinical pathway.

Both for cardiology and oncology, only short term activity and health outcome data can be already available. In addition, even if some national surveys were conducted, generalizability of provided data must be proven and more detailed data collected. Studies should be extended in time, in order to collect real world evidence (RWE) on the long-term consequences of the COVID-19 outbreak on patients affected by other diseases.

However, available studies already provide useful and relevant results toward planning the new Italian NHS out of the first phase of the emergency. First of all, a better communication approach should be adopted so that patients in critical conditions do not avoid seeking medical attention, therefore, putting their lives at risk, as data in cardiology has already demonstrated. Furthermore, campaigns that aim to increase awareness of critical symptoms, even during emergencies, should not play a secondary role (7), as suggested in the analysis conducted on PCI centers in Campania.

As different approaches (hospital-based, territorial-based, or combined models) were taken by the Italian health system in order to respond to the COVID-19 emergency (3, 26), meanwhile, alternative organizational initiatives have been adopted to manage non-COVID patients. These alternative organizational solutions should be further investigated to support the NHS out of the seemingly less critical phase. Once our literature review is completed, an analysis of organizational models will be conducted covering the most relevant areas for the Italian “Core Benefit Package of Healthcare Services” (so called LEA—Livelli Essenziali di Assistenza). This kind of analysis will be necessary, in order to redefine the capacity and priorities of NHS in recuperating “unprovided care” during the COVID-19 outbreak. Additional factors to consider will be NHS decentralization and regional variability, as well as pre-existing horizontal fragmentation and continuity of care (27).

Some data is available both in cardiology (Table 1) and oncology (Table 2) on how single hospitals adapt their technological and human resources to the emergency and related preliminary results. In particular, preliminary data in cardiology were collected on how a hub-and-spoke model performed in Lombardy (9, 22). While, for oncology, a national survey (13) collected preliminary evidence on the activation (only in 19% of the 29 planned cases) and efficiency of oncology hub-and-spoke programs during the emergency. Only one study (18) simulated how recommendations for prioritizing urologic surgeries could impact the activity of high-volume academic centers. Effectiveness of models of care, internal hospital protocols, and prioritization criteria should be investigated, taking into consideration the local diffusion and evolution of COVID-19.

Ongoing telemedicine initiatives, which are promising in some local experiences (17, 20), require a better coordinated approach and clearer guidelines. In its weekly Instant Report dedicated to COVID-19, ALTEMS dedicated a special section to digital health solutions adopted at regional and local levels to support healthcare services and deal with the COVID-19 outbreak.

Our analysis did not focus on clinical research, but several preliminary data on delays and restrictions in clinical trials are available (17). Pragmatic steps to minimize impact on trials, as suggested in (25), had been taken. Remote management of treatments, remote meeting with other centers and delivery of treatment directly to patients or pharmacy were solutions adopted in Emilia Romagna (17).

Finally, healthcare professionals remain a key resource for the NHS. The COVID-19 outbreak determined an additional workload for them, including crisis unit meetings (19), the need to learn new and different skills in the case of COVID-dedicated staff, the need to adapt a new organization in a short timeframe, the need to learn new ways to provide assistance (e.g., telemedicine, remote multidisciplinary meetings) (20), and so on. Associated with this was a shortage of specific profiles, such as intensivists. In addition, exposure to COVID-19 was and is a serious professional risk (28), as is demonstrated by the more than 29,000 positive COVID-19 cases among healthcare professionals and more than 160 and 40 deaths among clinicians and nurses, respectively, on a national level. In Torzilli et al. (13) was reported that in 33% of the departments for oncological surgery, which responded to the survey, at least one surgeon became COVID+. They represented up to 38% of the working power of the teams. While according to the survey conducted by the Italian Association of Radiotherapy and Clinical Oncology (AIRO) (12), 45% of centers had more than one staff persons in quarantine and 8.8% of centers had more than 5 units off.

## Conclusions

The SARS-CoV-2 (COVID-19) pandemic produced a dramatic impact in terms of deaths. But the actual impact on the health status of the population can only be measured if we look at it from a broader perspective. The reduction in the accessibility of non-COVID patients to healthcare services is a side effect of the COVID-19 outbreak, having a potential impact on the health of the population in the short and long term. Our paper has shed light on the short-term, and the indirect impact of COVID-19 for oncologic and cardiologic patients in Italy. The results of our literature review suggest that the emergency has reduced the accessibility of patients to hospitals and other healthcare services, and that it is already possible to identify a negative effect on clinical outcomes.

This evidence has implications for regional and national health policies and planning. In other words, the COVID-19 outbreak has reduced the capacity of the NHS to ensure the “Core Benefit Package of Healthcare Services” (so called LEA—Livelli Essenziali di Assistenza) that, by legislation, should be provided via healthcare organizations under the coordination of 21 Regional Health Authorities.

The Ministry of Health has recently (1 June 2020) issued “Guidelines for the progressive re-activation of planned healthcare services considered deferrable during the COVID-19 emergency” to regions, for both outpatient and inpatient care. The implementation of these guidelines by the regions is absolutely crucial in preventing a progressive extension of waiting lists for patients whose clinical conditions are worsening. The acceleration of medical cycles, and a more intense use of diagnostic technologies and operating theaters, could be the solutions to implement in facing this new challenge. Nevertheless, the availability of extra resources (e.g., availability of doctors and nurses) is necessary to increase the productivity of the healthcare system in this situation.

The lack of this acceleration could have long term implications in terms of clinical outcomes for individual patients, with a deterioration of basic health status indicators, such as mortality and disability.

## Author Contributions

RDB and AC contributed to the design, implementation of the research, to the analysis of the results, and to the writing of the manuscript. All authors contributed to the article and approved the submitted version.

## Conflict of Interest

The authors declare that the research was conducted in the absence of any commercial or financial relationships that could be construed as a potential conflict of interest.
