# Supplementary material for: Impact of SARS-CoV-2 on Provided Healthcare. Evidence From the Emergency Phase in Italy
Source: Front Public Health. 2020 Nov 23;8:583583. doi: 10.3389/fpubh.2020.583583 (PMC7719765; doi:10.3389/fpubh.2020.583583)
Supplement: Supplementary file 1 [file Table_1.DOCX]

Supplementary Material

# Supplementary Tables

Table A1. Cardiology: selected studies

| **Reference** | **De Rosa 2020** | **De Filippo 2020** | **Piccolo 2020** | **Cosentino 2020** | **Mazzone 2020** |
| --- | --- | --- | --- | --- | --- |
| NHS level | National (n=54 centres) | Multicentre (n=13)/Regional (n=5) | Regional (n=20 PCI centre) | Hospital | Hospital |
| **Clinical variables** |  |  |  |  |  |
| STEMI - NSTEMI | Yes | Yes | Yes | Yes |  |
| Unstable angina | Yes | Yes |  |  |  |
| Heart failure | Yes |  |  |  |  |
| Atrial fibrillation | Yes |  |  |  |  |
| Coronary angiography/PCI | Yes |  | Yes | Yes |  |
| Mortality | Yes |  |  | Yes |  |
| COVID positive patients | Yes |  |  | Yes |  |
| **Organizational variables** |  |  |  |  |  |
| Organisational changes |  |  |  | Yes | Yes |
| Prioritisation criteria |  |  |  |  | Yes |

Table A2. Oncology: selected national studies.

| **Reference** | **Costantini 2020** | **Indini 2020** | **Jereczek-Fossa 2000** | **Torzilli 2020** | **Progetto «La salute:**  **un bene da difendere”** | **Lambertini 2020** |
| --- | --- | --- | --- | --- | --- | --- |
| NHS level | National | National | National | National | National | National |
| **Clinical variables** |  |  |  |  |  |  |
| Surgical procedures |  |  |  | Yes |  |  |
| COVID diagnosis |  | Yes | Yes | Yes |  |  |
| **Organisational variables** |  |  |  |  |  |  |
| Clinical activity volumes | Yes |  | Yes | Yes |  |  |
| Bed availability |  |  |  | Yes |  |  |
| Personnel availability | Yes | Yes | Yes |  |  |  |
| Organisational changes | Yes | Yes | Yes | Yes |  | Yes |
| Prioritisation criteria |  |  | Yes |  |  | Yes |
| Telemedicine |  |  |  |  |  |  |
| Preventive measures |  | Yes | Yes |  |  |  |
| **Patient perspective** |  |  |  |  |  |  |
| Perception |  |  |  |  | Yes |  |
| Access to care |  |  |  |  | Yes |  |
| Health outcomes |  |  |  |  | Yes |  |

Table A3, Oncology: selected regional studies.

| **Reference** | **Casanova 2020** | **Brandes 2020** | **Campi 2020** |
| --- | --- | --- | --- |
| NHS level | Regional | Regional | Hospitals |
| **Clinical variables** |  |  |  |
| Surgical procedures |  |  | Yes |
| COVID diagnosis |  |  |  |
| **Organisational variables** |  |  |  |
| Clinical activity volumes |  | Yes |  |
| Bed availability |  | Yes |  |
| Personnel availability |  | Yes |  |
| Organisational changes |  | Yes |  |
| Prioritisation criteria |  | Yes |  |
| Telemedicine |  |  |  |
| Preventive measures |  |  |  |
| **Patient perspective** |  |  |  |
| Perception | Yes |  |  |
| Access to care |  |  |  |
| Health outcomes |  |  |  |

Table A4. Oncology: selected studies focused on a single centre/hospital experience.

| **Reference** | **Balduzzi 2020** | **Bongiovanni 2020** | **Kengli 2020** | **Vicino 2020** | **Montesi 2000** | **Pezzulla 2020** |
| --- | --- | --- | --- | --- | --- | --- |
| NHS level | Hospital | Hospital | Hospital | Hospital | Hospital | Hospital |
| **Clinical variables** |  |  |  |  |  |  |
| Surgical procedures |  |  |  |  |  |  |
| COVID diagnosis |  |  |  |  |  |  |
| **Organisational variables** |  |  |  |  |  |  |
| Clinical activity volumes |  | Yes |  | Yes | Yes |  |
| Bed availability |  |  |  |  |  |  |
| Personnel availability |  |  |  |  |  |  |
| Organisational changes | Yes | Yes | Yes | Yes | Yes | Yes |
| Prioritisation criteria | Yes |  | Yes |  | Yes |  |
| Telemedicine |  | Yes |  |  |  |  |
| Preventive measures |  |  |  |  |  |  |
| **Patient perspective** |  |  |  |  |  |  |
| Perception |  |  |  |  |  |  |
| Access to care |  |  |  |  |  |  |
| Health outcomes |  |  |  |  |  |  |
